# Notes on the genus Apteroloma of China with description of a new species (Coleoptera, Agyrtidae)

**DOI:** 10.3897/zookeys.124.1774

**Published:** 2011-08-18

**Authors:** Liang Tang, Li-Zhen Li, Jan Růžička

**Affiliations:** 1 Department of Biology, Shanghai Normal University, 100 Guilin Road, 1st Educational Building 323 Room, Shanghai, 200234 P. R. China; 2 Department of Ecology, Faculty of Environmental Sciences, Czech University of Life Sciences in Prague, Kamýcká 129, CZ-165 21 Praha 6, Czech Republic

**Keywords:** Agyrtidae, *Apteroloma*, China, Coleoptera, new species, Palaearctic region, taxonomy

## Abstract

A new species, *Apteroloma zhejiangense* **sp. n.**, is described from Zhejiang Province, China. The habitus and sexual characters of the new species are illustrated. *Apteroloma jinfo* Růžička, is reported for the first time from China: Hubei and Hunan Provinces, and *Apteroloma potanini* (Semenov, 1893) from Ningxia and Henan Provinces. Presence of *Apteroloma kozlovi* Semenov-Tian-Shanskij & Znojko in Semenov-Tian-Shanskij, 1932 in South Korea is confirmed based on re-examined material.

## Introduction

*Apteroloma* Hatch, 1927 is a large genus of Agyrtidae with 29 species in the world and 8 species in China. The Chinese species of the genus were recently revised by Růžička et al. (2004) and Růžička and Pütz (2009).

In this paper, a new species from Zhejiang Province is described which considerably expands the distribution of the genus to East China. New province records of several *Apteroloma* species from China are provided and *Apteroloma kozlovi* from South Korea is confirmed based on re-examined material.

## Material and methods

For examination of genitalia, the last two male abdominal segments and last female abdominal segment were detached from the body after softening in hot water. The aedeagus together with other dissected pieces were mounted in Euparal (Chroma Geselschaft Schmidt, Koengen, Germany) on plastic slides. Photos of sexual characters were taken with Canon G7 attached to Olympus SZX 16 stereomicroscope; habitus photos were taken with a Canon macro photo lens MP-E 65mm attached to Canon EOS40D camera.

The measurements are abbreviated as follows:

EL	length of elytra (measured from caudal tip of scutellum to elytral apex)

EW	combined maximum width of elytra

PBW	pronotal basal width

PML	pronotal medial length

PMW	pronotal maximum width

TBL	total body length.

The specimens treated in this study are deposited in the following public and private collections:

JRUC	Jan Růžička collection, Praha, Czech Republic

MPEC	Michel Perreau collection, Paris, France

NMPC	National Museum, Praha, Czech Republic (Jiří Hájek)

NSMT	National Science Museum, Tokyo (Shûhei Nomura)

SHNU	Department of Biology, Shanghai Normal University, P. R. China (Liang Tang)

## Taxonomy

### Apteroloma zhejiangense

Tang, Li & Růžička sp. n.

urn:lsid:zoobank.org:act:115949B4-715B-4D54-8634-A515CED52E1D

http://species-id.net/wiki/Apteroloma_zhejiangense

Figs 1, 2 5–8 

#### Type locality.

China, Zhejiang Province, Anji County, Longwangshan N. R. [ca. 30°22'N, 119°30'E ].

#### Type material.

 **China: Zhejiang Province:** **Holotype.** Male, glued on a card, with genitalia mounted on a plastic slide in Euparal, with labels as follows: “Mt. Longwangshan N. R. / 950–1200m / Zhejiang Prov. / 25-IV-2004 / Liang Tang leg.” “Holotype / *Apteroloma zhejiangense* / Tang, Li & Růžička det. 2011” [red handwritten label] (SHNU) and “Paratype / *Apteroloma zhejiangense* / Tang, Li & Růžička det. 2011” [yellow printed label] for the rest of the type series. **Paratypes.** 1 male and 1 female, Longwanshan, 3.X.2003, Hu & Tang leg. (SHNU); 1 male, Longwanshan, 950–1200m, 25.IV.2004, Liang Tang leg. (SHNU); 1 male, Longwanshan, 300–500m, 26.IV.2004, Li & Hu leg. (SHNU); 4 males and 1 female, Longwangshan, 950–1200m, 25.IV.2004, Liang Tang, Jia-Yao Hu & Li-Long Zhu leg. (SHNU); 6 males and 7 females, Longwangshan N. R., alt. 950–1200m, 25.IV.2006, Liang Tang, Rui-Fen Lin, Xin Yuan, Jin-Wen Li & Shan-Jia Shen leg. (1 pair in JRUC, rest in SHNU).

#### Description.

 Measurements of the male holotype: TBL=7.3 mm, PMW/PML=1.49, PMW/PBW 1.19, EL/EW 1.33, EW/PMW 1.53.

Body large, 6.7–7.7 mm in length; dorsum in mature specimens dark brown; antennae, mouthparts, lateral portion of pronotum and legs uniformly ferruginous; dorsal surface shiny, with fine transverse microsculpture (pronotum discally with nearly isodiametric microsculpture); pronotum and elytra with scattered short erect setation; each mandible with two large acute teeth on inner edge before apex.

**Figures 1–4.**
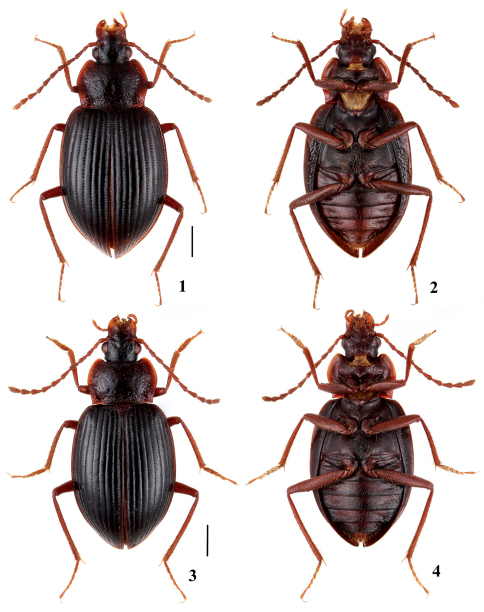
Adult of *Apteroloma* in dorsal and ventral view. **1, 2** *Apteroloma zhejiangense* sp. n. (paratype female) **3, 4** *Apteroloma jinfo* (male from Hubei). Scales = 1 mm.

Pronotum widest in middle; anterior margin weakly emarginate; lateral margins distinctly bordered; weakly explanate; sides flat, only moderately raised and weakly sinuate posteriorly (Fig. 1); base wide, without impressions. Disc with scattered fine punctures, lateral and posterior areas heavily, densely punctured.

Elytra broadly oval. Each elytron with nine regular striae, third stria with ca. 49–59 medium-sized punctures; lateral margin smooth, without serration; epipleural keel narrow. Elytral epipleura with strong and dense punctures (Fig. 2). Metathoracic wings fully developed.

**Figures 5–12.**
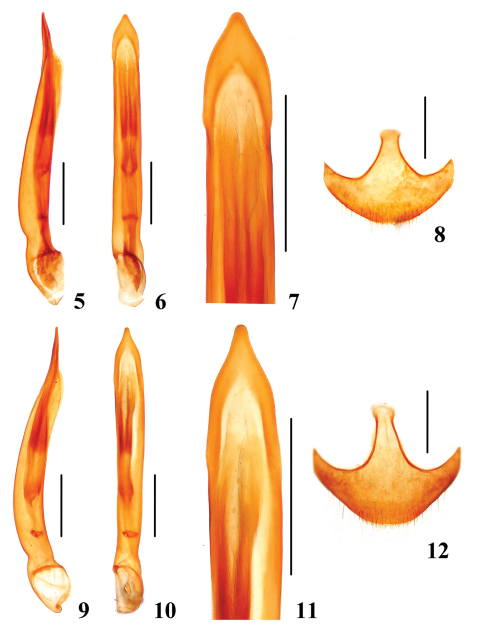
Sexual structures of *Apteroloma*. **5–8** *Apteroloma zhejiangense* sp. n. **9–12** *Apteroloma jinfo* (Hubei); **5, 9** aedeagus laterally **6, 10** aedeagus ventrally **7, 11** apex of aedeagus ventrally **8, 12** female ventrite VIII ventrally. Scales = 0.5 mm.

Male. Aedeagus evenly rounded with elongate, straight apex in lateral view (Fig. 5); sides before apex broadened, with blunt tip in dorsal view (Figs 6, 7).

Female. Ventrite VIII regularly rounded posteriorly, spiculum ventrale short, narrow and truncate anteriorly (Fig. 8). Ovipositor with transverse valvifer without setae; triangular, heavily sclerotized coxite bearing numerous setae; and stylus modified into strongly curved, apically glabrous scrapers.

#### Diagnosis.

 The new *Apteroloma* species, is most similar to *Apteroloma jinfo*, both species share the following characters in combination: body large (6.7–7.7 mm in length), dorsum dark brown with uniformly ferruginous appendages (Figs 1, 3), pronotum with wide base (Figs 1, 3), aedeagus with elongate apex in lateral view (Figs 5, 9) and sides before apex only slightly broadened in dorsal view (Figs 7, 11), female ventrite VIII with narrow spiculum ventrale (Figs 8, 12).

The recently published key of Chinese *Apteroloma* (Růžička and Pütz 2009) should be modified at the couplet #5 as follows to accommodate the new species:

**Table:** 

5	Large species (body length 6.7–7.7 mm)	5a
–	Small species (body length 5.4–6.8 mm)	6
5a	Pronotum with sparse punctation discally, sides distinctly sinuate posteriorly (Fig. 3). Elytra with fine punctation (third stria with 62–67 punctures, Fig. 3). Elytral epipleura with fine punctures (Fig. 4). Aedeagus with narrow, elongate tip (Fig. 11). Ventrite VIII with spiculum ventrale elongate (Fig. 12). Central China (Chongqing, Hubei, Hunan) (Fig. 13)	*Apteroloma jinfo* Růžička, Schneider & Háva, 2004
–	Pronotum with dense punctation discally, sides weakly sinuate posteriorly (Fig. 1). Elytra with strong punctation (third stria with 49–59 punctures, Fig. 1). Elytral epipleura with strong punctures (Fig. 2). Aedeagus with subapically broadly rounded, blunt tip (Fig. 7). Ventrite VIII with spiculum ventrale short (Fig. 8). Eastern China (Zhejiang) (Fig. 13)	*Apteroloma zhejiangense* sp. n.

#### Etymology.

 Adjective; named after Zhejiang Province, where the new species was discovered.

#### Distribution.

**Figure 13.**
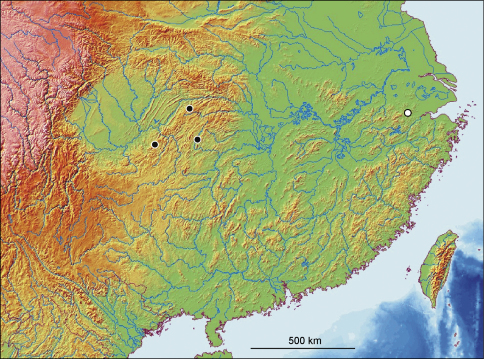
Distribution of *Apteroloma zhejiangense* sp. n. (empty circle) and *Apteroloma jinfo* (filled circles) in China.

 China (Zhejiang) (Fig. 13). So far known only from the type locality.

#### Bionomics.

 All specimens of the new species were collected by sifting leaf litters in broad-leaved forest, at altitudes 300–1200 m.

### Apteroloma jinfo

Růžička, Schneider & Háva, 2004

http://species-id.net/wiki/Apteroloma_jinfo

Figs 3, 4 9–12

Apteroloma jinfo Růžička et al. 2004: 116.

#### Material examined.

 **China: Chongqing municipality:** data of type series as inRůžička et al. (2004: 116); **Hubei Province:** 8 males and 8 females, Wufeng County, Houhe N. R. [ca. 30°33'N, 108°55'E ], 30.IV.2004, Li-Zhen Li leg. (1 pair in JRUC, rest in SHNU); **Hunan Province:** 3 males, Longshan Xian, Huoyan Xiang, Huoyan, entrance to Feihu Dong cave [ca. 29°12'N, 109°18'E ], 20.IX.1997, Y. Nishikawa leg. (1 male JRUC, 2 males NSMT).

#### Variation.

 Aedeagus has distinctly triangular, sub-sinuate apex in dorsal view in specimens from the type locality (Růžička et al. 2004: 117, Fig. 3), and straight apex with narrower tip in dorsal view in males from Hubei and Hunan Provinces (Fig. 11). Posterior margin of female ventrite VIII in specimens of Hubei Province is regularly rounded (Fig. 12), not distinctly truncate as observed in specimens from the type locality (Růžička et al. 2004: 117, Fig. 10). However, we consider variability of both characters to fall within variability range of the same species.

#### Distribution.

 China (Chongqing, Hubei, Hunan) (Fig. 13). The type locality (Jinfo Shan) was erroneously located in Sichuan Province by Růžička et al. (2004) and Růžička & Pütz (2009). First records from Hubei and Hunan Provinces.

### Apteroloma potanini

(Semenov, 1893)

http://species-id.net/wiki/Apteroloma_potanini

Pteroloma potanini Semenov 1893: 338.
Apteroloma potanini : Semenov-Tian-Shanskij 1932: 338; Růžička et al. 2004: 122; Růžička and Pütz 2009: 642.

#### Material examined.

 **China: Ningxia Province:** 1 male, Longde County, Qingliang, 35°36'19"N, 106°11'02"E , alt. 2400m, 26.VI.2008, Zi-Wei Yin leg. (SHNU); 1 male, Jingyuan County, Dongshanpo Forestry, Haizigou, 35°37'13"N, 106°15'42"E , alt. 2300m, 24. VI. 2008, Zi-Wei Yin leg. (SHNU); **Henan Province:** 2 specimens: Quanbaoshan, Xiong’er Shan, 34°07'N, 111°25'E , alt. 1700m, 23.–24.V.2010, broadleaved forest, beside forest path, first specimen in fly and second one at a big stone covered by moss, M. Perreau det. (MPEC); 1 specimen: same data, but alt. 1850m, pitfall trap (baited with beer and antifreeze) (MPEC).

#### Distribution.

 China (Sichuan, Shaanxi, Gansu, Ningxia, Hubei, Hebei, Henan). First records from Ningxia and Henan Provinces.

### Apteroloma kozlovi

Semenov-Tian-Shanskij & Znojko, 1932

http://species-id.net/wiki/Apteroloma_kozlovi

Apteroloma kozlovi  Semenov-Tian-Shanskij & Znojko in Semenov-Tian-Shanskij 1932: 341.
Garytes coreanus Mroczkowski 1966: 434 (synonymy by Růžička et al. (2004)).

#### Material examined.

 **China: Shaanxi Province:** 1 male, 110 km NNE Xian, Huayin vill., Hua Shan Mt., 34°29.5'N, 110°05.1'E , alt. 1275m, 8.–9.V.2011, granite cliff, valley of small brook, gravel stones close to water, M. Balke & J. Hájek leg. (NMPC); **South Korea:** 1 female, GanWeon-do, ChungChong [= Gangwon-do province, Chuncheon, ca. 37°52'N, 127°44'E ], 17.V.1984, K. Morimoto leg. (NSMT); 2 males, KangWeon-do, HongCheon-gun, Pupan-myon [= Gangwon-do province, Hoengseong-gun county (centroid ca. 37°29'N, 127°59'E ), Pupan myeon township (not located)], 20.V.1992, S. Nomura leg. (NSMT); 4 males, 2 females, Gyeongsamnam-do, Macheong-meon, Samjeong-li [= Gyeongsangnam-do province, Macheong-myeon township, Samjeong-ri village, ca. 35°21.5'N, 128°56.7'E ], 9.V.1991, S. Nomura leg. (NSMT).

#### Note.

 The examined material from South Korea (reported as *Apteroloma potanini* by Nomura and Lee 1993) is verified here to belong to *Apteroloma kozlovi*. *Apteroloma kozlovi* was considered as junior subjective synonym of *Apteroloma potanini* by Schawaller (1991), and resurrected as a valid species only by Růžička et al. (2004).

#### Distribution.

 China (Qinghai, Shaanxi, Shanxi, Hebei, Beijing); North Korea; South Korea.

## Supplementary Material

XML Treatment for Apteroloma zhejiangense

XML Treatment for Apteroloma jinfo

XML Treatment for Apteroloma potanini

XML Treatment for Apteroloma kozlovi
